# Illness perception and insomnia among patients with maintenance hemodialysis: a moderated mediation model of fear of progression and self-compassion

**DOI:** 10.1080/0886022X.2026.2670075

**Published:** 2026-07-21

**Authors:** Yalan Huang, Mengting Chen, Shanshan Zhong, Yuan Liu, Ruisi Xu, Yongguang Li, Kangping Li, Yu Wei, Xiaoxiao Luo

**Affiliations:** a Outpatient Department, Yunnan Provincial Corps Hospital of Chinese People’s Armed Police Force, Kunming, China; b Department of Clinical Nutrition, Chongqing University Cancer Hospital, Chongqing University, Chongqing, China; c Renji Hospital, School of Medicine, Chongqing University (The Fifth People’s Hospital of Chongqing), Chongqing, China; d Intensive Care Unit, Bethune International Peace Hospital, Shijiazhuang, China; e Chongqing Rongchang District Jinshui Aishen Hemodialysis Center Co., Ltd.

**Keywords:** Maintenance hemodialysis patients, insomnia, illness perception, fear of progression, self-compassion

## Abstract

This research examined the impact of illness perception, fear of progression, and self-compassion on insomnia among maintenance hemodialysis patients in China. A cross-sectional study was conducted in Chongqing from January 2025 to May 2025. The Brief Illness Perception Questionnaire, Athens Insomnia Scale, Fear of Progression Questionnaire-Short Form, and Self-Compassion Scale-Short Form were evaluated in 332 maintenance hemodialysis patients. This research found that 55.72% of the patients suffered from insomnia. Insomnia showed a positive relationship with illness perception (*r* = 0.629, *p* < 0.01) and fear of progression (*r* = 0.479, *p* < 0.01), but a negative relationship with self-compassion (*r* = −0.451, *p* < 0.01). The mediating role of fear of progression in the relationship between illness perception and insomnia accounted for 17.5% of the total impact. Analysis of simple slopes revealed that illness perception was a significant predictor of fear of progression at both low (*B* = 0.477, *t* = 5.131, and *p* < 0.001) and high (*B* = 0.732, *t* = 8.187, and *p* < 0.001) self-compassion levels. Our study’s findings contribute to the understanding of factors affecting insomnia among maintenance hemodialysis patients and offer a theoretical foundation and practical advice for improving their sleep quality.

## LAY (PLAIN LANGUAGE) SUMMARY

Patients receiving regular hemodialysis often struggle with poor sleep. In our study of 332 dialysis patients in Chongqing, China, more than half (55.7%) reported significant insomnia. This sleep disturbance can reduce quality of life and worsen health outcomes. This study looked at how patients’ views about their illness (illness perception), worries about their disease getting worse (fear of progression), and their ability to be kind to themselves (self-compassion) affect sleep problems. We found that more negative views about kidney disease were related to worse sleep, partly because of increased fear of disease progression. We also found that patients who were more self-compassionate were less affected by negative illness perceptions. Implementing strategies like short self-compassion exercises or cognitive-behavioral therapy for insomnia (CBT-I) during dialysis sessions might enhance sleep quality in in this vulnerable population. Screening for insomnia and psychological distress should be a routine part of dialysis care.

## KEY MESSAGES

**What is known:** Insomnia is highly prevalent in maintenance hemodialysis (MHD) patients and is linked to poor quality of life and adverse clinical outcomes. The relationship between psychological factors like illness perception and self-compassion with sleep quality in chronic illness is known, but their interaction in insomnia related to hemodialysis is not well comprehended.

**This study adds:** Fear of disease progression partially mediates the relationship between illness perception and insomnia, while self-compassion significantly moderates the direct pathway from negative illness perception to insomnia.

**Potential impact:** Targeted psychological interventions aimed at reducing fear of progression and enhancing self-compassion may help alleviate insomnia among MHD patients, supporting integrated clinical care strategies.

## Background

Maintenance hemodialysis (MHD) is a life-sustaining treatment for end-stage renal disease (ESRD), but it is accompanied by a high burden of sleep disturbances, particularly insomnia. Epidemiological research has found that between 60.9 and 67.3% of MHD patients experience insomnia [1]. The high occurrence of insomnia in MHD patients is closely linked to negative outcomes such as depression, heart issues, and a lower quality of life related to health [2]. The causes of insomnia in MHD are multifaceted, with intricate interactions between biological and psychological elements [3]. From a biological perspective, the buildup of uremic toxins interferes with circadian rhythms and melatonin production. Meanwhile, hemodialysis causes sleep disturbances due to its weekly schedule and frequent naps during treatment [4]. Sleep disturbances are further worsened by comorbid sleep disorders like sleep apnea, which affects 53.2% of MHD patients [5]. Despite the rising awareness of psychological elements, the impact of cognitive and emotional factors is still not fully investigated in this context.

Illness perception, defined as individuals’ cognitive and emotional understanding of the disease, plays a critical role in influencing health outcomes in chronic illnesses [6]. Previous studies on MHD patients have associated negative perceptions of illness with social isolation and decreased adherence to treatment, but the specific connection to insomnia has not been extensively studied [7]. Recent findings indicate that illness perceptions might trigger a series of psychological reactions, such as fear of progression (FoP), potentially influencing sleep issues [8].

Fear of progression, characterized by excessive anxiety about disease deterioration or recurrence, has emerged as a significant psychological stressor in chronic illnesses. Meta-analysis data supports that FoP serves as a transdiagnostic mediator connecting physical symptoms to negative mental health outcomes [9,10]. In individuals undergoing MHD, fear of progression might develop due to the need to constantly monitor uremic symptoms, anxiety over potential treatment issues and the uncertain path of the disease [11]. Studies have previously demonstrated that the fear of progression is significantly associated with sleep quality in chronic conditions [12]. The FoP can lead to hyperarousal, excessive thinking, and anxiety related to sleep, which are crucial mechanisms in the development of insomnia. However, no studies have specifically investigated whether FoP mediates the association between illness perception and insomnia in this population.

Self-compassion involves being gentle and caring toward oneself during difficult or stressful times, encompassing three aspects: self-kindness, common humanity, and mindfulness [13,14]. Self-compassion is viewed as a beneficial psychological trait, and it has been shown to mitigate the negative effects of stress on mental health [15,16]. While direct research on self-compassion in MHD is limited, a qualitative study studies indicate that higher self-compassion correlates with better psychological well-being in hemodialysis patients [17]. Research indicates that self-compassion lessens the effects of stress on sleep by decreasing rumination and anxiety related to sleep [18]. Additionally, studies have found that self-compassion serves as a protective element against poor sleep, inversely related to insomnia [19]. Mindfulness-based interventions targeting self-compassion have shown promise in reducing postpartum depression among a group of symptomatic pregnant women, supporting the feasibility of such constructs in renal care [20]. Thus, considering the numerous studies showing the beneficial protective effects of self-compassion, we can hypothesize that self-compassion might influence the relationship between illness perception, fear of progression and insomnia among patients with maintenance hemodialysis.

The conceptual framework guiding this study is Leventhal’s Common-Sense Model of Self-Regulation (CSM). According to the CSM, when individuals face a health threat such as end-stage renal disease, they form illness perceptions. These perceptions drive coping behaviors and emotional responses, which subsequently influence health outcomes, including sleep disturbance. In the context of maintenance hemodialysis, negative illness perceptions are hypothesized to generate fear of progression (FoP). FoP leads to cognitive hyperarousal and rumination, which are essential mechanisms in causing insomnia. Within this pathway, self-compassion is posited as a moderating buffer.

Despite advances in understanding sleep disturbances in MHD, there are still critical gaps. Current studies have mainly concentrated on biological determinants or individual psychological factors, with little emphasis on the interaction between illness perception, fear of progression and self-compassion in affecting insomnia. A model that fully integrates these factors is essential for the development of targeted interventions. Based on prior research, this study aimed to assess the effects of illness perception, fear of progression and self-compassion on insomnia in individuals undergoing MHD. Our hypothesis was that (1) illness perception is positively associated with insomnia; (2) fear of progression mediates the relationship between illness perception and insomnia; (3) self-compassion moderated the relationship between illness perception and insomnia.

## Materials and methods

### Design, participants and procedure

This research was a cross-sectional study, including 332 MHD patients who received hemodialysis between January 2025 and May 2025 at Chongqing Yongchuan Kinshui Blood Purification Center. The criteria for inclusion were: (1) being 18 years or older, (2) receiving regular hemodialysis for over three months, (3) no problems related to cognitive or communication abilities, and (4) having signed an informed consent form. Patients who qualified for the study were asked to fill out a self-reported questionnaire and received consistent instructions from a trained researcher. Patients were informed about the study’s purpose and procedures before the questionnaire started. The researchers gathered responses through an online questionnaire. Besides, the researcher assisted individuals who couldn’t fill out the questionnaire on their own by reading the questions aloud and objectively noting their responses. The questionnaire was conducted over a period of 20 to 30 min. After participants complete the questionnaire, researchers will promptly review it. Any incomplete responses will be requested to be completed immediately.

The ethics committee of the Fifth People’s Hospital of Chongqing approved this study (approval number 2025CQSDWRMYYEC-lw004). A clinical trial number does not apply. The methods were implemented in compliance with the rules and guidelines of the Declaration of Helsinki. Acquired permission from the hospital to distribute research questionnaires and conducted surveys anonymously. Prior to taking part in the study, every participant voluntarily provided written informed consent. The study invited 351 MHD patients, and 332 of them agreed to participate, achieving a response rate of 94.59%.

### Demographics and clinical data

Trained nurses gathered demographic and clinical information through structured questionnaires during face-to-face interviews with MHD patients waiting for hemodialysis. The subsequent details were documented: sex, age, educational level, marital status, medical payment methods, comorbidities, duration of dialysis, height, weight, monthly household income, employment status and smoke. Comorbidities including hypertension, diabetes, cardiovascular disease, and other chronic conditions were documented. Medications with potential effects on sleep, as well as dialysis duration and treatment intensity, were also recorded.

### Illness perception

This investigation employed the Chinese version of the brief illness perception questionnaire (BIPQ) to analyze the cognitive and emotional views of an illness among MHD patients [21]. The survey included 8 questions, with scores ranging from 0 to 80. Elevated scores indicated a greater perceived threat of illness and more negative patient perspectives. The scale, now regularly employed with hemodialysis patients, has a Cronbach’s alpha coefficient of 0.757 [22]. The scale in this research exhibited a Cronbach’s α coefficient of 0.782.

### Insomnia

We used the Athens Insomnia Scale (AIS) to measure the Insomnia in MHD patients [23]. There are 8 items in the AIS and each one scored from 0 to 3. The overall score of the scale is determined by adding up the scores of each item, indicating a spectrum from none to severe. Scores less than 4 imply no sleep disorder, scores from 4 to 6 suggest potential insomnia, and scores greater than 6 indicate insomnia. The Chinese version of AIS has demonstrated strong validation [24], with a Cronbach’s alpha of 0.693 in this research.

### Fear of progression

The assessment of FOP was conducted using the Fear of Progression Questionnaire-Short Form (FOP-Q-SF), which Mehert adapted from the FoP-Q [25]. The assessment includes 12 self-reported items and covers two dimensions: physical health and social and family aspects. Every item is rated on a scale from 1 to 5, where a higher score indicates a greater level of fear. A score of 34 points signifies dysfunctional FOP. The Chinese FOP-Q-SF version has demonstrated strong validation [26]. The current research found a Cronbach’s α coefficient of 0.869 for the scale, suggesting good internal consistency.

### Self-compassion

We assess self-compassion *via* the Self-Compassion Scale-Short Form (SCS-SF) [27]. The SCS-SF consists of 12 items designed to measure six elements of self-compassion: self-kindness, self-judgment, common humanity, isolation, mindfulness and over-identification. A 5-point Likert-type scale was used to score the items, with 1 representing ‘almost never’ and 5 representing ‘almost always’. Higher levels of self-compassion are indicated by a higher score. In this study, the overall reliability coefficient for the total score was 0.950, with subscale values ranging from 0.685 to 0.794.

### Data analysis

SPSS (version 26.0) and the Hayes SPSS macro program PROCESS (version 3.2) were utilized to manage and analyze the data. Initially, categorical variables were described using frequency and percentage. Continuous variables were described using mean and standard deviation (SD). Pearson correlation analysis was employed to examine the connections between the study variables. All demographic and clinical variables listed in Table 1 were entered as covariates in Step 1 of the hierarchical regression model to control for their potential confounding effects. The analytical strategy proceeded in two primary steps. First, we conducted the mediation analysis by using PROCESS Model 4 to examine whether fear of progression mediated the association between illness perception and insomnia. The indirect effect was estimated with bias-corrected bootstrap 95% confidence intervals based on 5,000 resamples. Second, moderation of the direct effect was tested using hierarchical multiple regression to determine whether self-compassion moderated the relationship between illness perception and insomnia. The interaction term BIPQ × SCS was calculated after mean-centering the constituent variables. Simple slopes were evaluated at low (−1 SD) and high (+1 SD) levels of self-compassion. Two-sided tests were used for all statistical analyses, and P values less than 0.05 were considered statistically significant.

**Table 1.** Participant characteristics.

Variables	*N* (%)			Insomnia	t/F	*p*
Sex					1.223	0.484
Male	210 (63.25%)			7.83 ± 5.43		
Female	122 (37.89%)			8.25 ± 5.18		
Age					1.153	0.328
≤30	35 (10.54%)			8.31 ± 5.07		
21–45	92 (27.71%)			7.13 ± 4.85		
46–60	144 (43.37%)			8.42 ± 5.55		
≥60	61 (18.37%)			8.07 ± 5.63		
Residence					0.626	0.430
Urban	197 (59.34%)			7.98 ± 5.30		
Rural	135 (40.66%)			7.99 ± 5.40		
Education					0.432	0.650
Junior high school or below	238 (71.69%)			7.98 ± 5.47		
Senior high school	69 (20.78%)			7.68 ± 4.85		
Junior college or above	25 (7.53%)			8.84 ± 5.38		
Marital status					0.086	0.968
Unmarried	49 (14.76%)			8.00 ± 4.96		
Married	239 (71.99%)			7.99 ± 5.38		
Divorced/	37 (11.14%)			8.11 ± 5.59		
Widowed	7 (2.11%)			7.00 ± 5.72		
Medical payment methods					5.746	0.004
Resident health insurance			146 (43.98%)	9.09 ± 5.61		
Employee medical insurance			164 (49.40%)	7.12 ± 4.96		
Others			22 (6.63%)	7.09 ± 5.04		
Comorbidities					0.313	0.731
No			34 (10.24%)	8.26 ± 6.06		
One or two			231 (69.58%)	8.07 ± 5.33		
Three or more			67 (20.18%)	7.54 ± 4.99		
Duration of dialysis (years)					0.952	0.416
Less than 2 years			63 (18.98%)	7.52 ± 4.96		
2–5 years			112 (33.73%)	7.68 ± 5.43		
6–10 years			104 (31.33%)	8.07 ± 5.39		
More than 10 years			53 (15.96%)	9.02 ± 5.43		
BMI (%)					2.543	0.080
Normal			193 (58.13%)	8.41 ± 5.28		
Underweight			33 (9.94%)	8.58 ± 4.93		
Overweight			106 (31.93%)	7.03 ± 5.46		
Monthly household income per capita					1.201	0.309
≤2000 RMB		201 (60.54%)		8.06 ± 5.25		
2000–3999 RMB			77 (23.19%)	7.95 ± 5.18		
4000–5999 RMB			34 (10.24%)	8.79 ± 6.10		
≥5000 RMB			20 (6.02%)	6.00 ± 5.32		
Employment status					0.892	0.373
Employed			81 (24.40%)	8.44 ± 5.24		
Unemployed			251 (75.60%)	7.84 ± 5.36		
Smoke					15.80	0.000
No			264 (79.52%)	7.40 ± 4.89		
Yes			68 (20.48%)	10.26 ± 6.31		

## Result

### Sample characteristics

In this study, 63.25% of the 332 participants were male. The mean age was 48.23 years (SD = 12.57), with ages ranging from 18 to 81. About 71.69% had education level junior below high school, 71.99% were married, 59.34% lived in urban, 20.18% suffered from 3 or more kinds of comorbidities, and 15.96% had been on dialysis for over 10 years. 49.40% of the participates had employee medical insurance. Regarding the monthly household income per capita, 201 participants (60.54%) were less than or equal to 2,000 RMB. Further details regarding the sample characteristics can be found in Table 1. The findings indicated that the method of medical payment (*t* = 5.746, *p* = 0.004) and smoking status (*t* = 15.80, *p* < 0.001) significantly influenced the prevalence of insomnia among patients undergoing maintenance hemodialysis. Patients enrolled in resident health insurance exhibited a significantly greater severity of insomnia compared to those with employee medical insurance or other forms of coverage. Additionally, patients who smoked demonstrated a higher degree of insomnia than their nonsmoking counterparts.

### Descriptive analysis

The study variables’ means and standard deviations are shown in Table 2. The mean score of illness perception was 45.78 (SD = 8.65). The average fear of progression score was 29.67 with a standard deviation of 10.79, and the prevalence rate was 33.43% (111 cases). The mean score of insomnia was 7.98 (SD = 5.33). About 22.89% (76 of 332) of those surveyed experienced suspected insomnia, and 55.72% (185 of 332) showed insomnia. The most prevalent comorbidities were hypertension (68.98%), diabetes mellitus (22.89%), and cardiovascular disease (8.74%). Anemia (hemoglobin < 100 g/L) was present in 33.73% of patients. The prevalence of restless legs syndrome is 12.35%. The patients taking sedative and hypnotic drugs account for 12.65%.

**Table 2.** Descriptive statistics and bivariate correlations of the major study variables (*n* = 332).

	M ± SD	BIPQ	FoP	SCS	AIS
BIPQ	45.78 ± 8.65	1			
FoP	29.67 ± 10.79	0.501**	1		
SCS	38.09 ± 5.99	−0.577**	−0.269**	1	
AIS	7.98 ± 5.33	0.629**	0.479**	−0.451**	1

Note: BIPQ, Brief Illness Perception Questionnaire; FoP, Fear of Progression; SCS, Self-compassion; AIS, Athens Insomnia Scale. Pearson correlation coefficients are reported. ** *p* < 0.01, * *p* < 0.05.

### Correlations of illness perception, fear of progression, self-compassion, and insomnia

Table 2 also displayed the outcomes of bivariate correlations among various primary variables. The illness perception was significantly positively correlated with fear of progression (*r* = 0.501, *p* < 0.01), insomnia (*r* = 0.629, *p* < 0.01), and negatively associated with self-compassion (*r* = −0.577, *p* < 0.01). There was a significant positive correlation between fear of progression and insomnia (*r* = 0.479), while it was negatively correlated with self-compassion (*r* = −0.269, *p* < 0.01). Self-compassion was significantly negatively associated with insomnia (*r* = −0.451, *p* < 0.01).

### Mediating effect analysis

Using Model 4 in the SPSS macro PROCESS software, the mediating effect analysis was performed to examine the impact of fear of progression on the link between illness perception and insomnia. According to Model 1 in Table 3, BIPQ was a significant positive predictor of AIS (*B* = 0.388, *t* = 14.679, *p* < 0.001, 95%CI [0.336,0.439]). Model 2 indicated that BIPQ was a significant positive predictor of FoP (*B* = 0.625, *t* = 10.508, *p* < 0.001, 95%CI [0.508,0.742]), and FoP significantly positively predicted AIS (*B* = 0.108, *t* = 4.570, *p* < 0.001, 95%CI [0.062,0.155]). Furthermore, as demonstrated in Model 3, the direct impact of BIPQ on AIS remained significant (*B* = 0.320, *t* = 10.795, and *p* < 0.001, 95%CI [0.261, 0.378]) even after including mediating variables. Moreover, the 95% CI from the bootstrap for both the direct effect of BIPQ on AIS and the mediating effect of FoP excluded 0, indicating a significant mediating effect. A moderated mediation model was supported. The indirect effect of illness perception on insomnia *via* fear of progression accounted for 17.5% of the total effect, and the direct path was moderated by self-compassion.

**Table 3.** Mediation analysis (*n* = 332).

	Model 1				Model 2				Model 3			
	(AIS)				(FoP)				(AIS)			
			Bootstrap 95%CI				Bootstrap 95%CI				Bootstrap 95%CI	
Variables	*β*	*t*	LLCI	ULCI	*β*	*t*	LLCI	ULCI	*β*	*t*	LLCI	ULCI
BIPQ	0.388	14.679	0.336	0.439	0.625	10.508	0.508	0.742	0.320	10.795	0.261	0.378
FoP									0.108	4.570	0.062	0.155
*R^2^*	0.395				0.251				0.431			
F	215.477***				110.427***				124.674***			

Note: BIPQ, Brief Illness Perception Questionnaire; FoP, Fear of Progression; AIS, Athens Insomnia Scale. ***p < 0.001.

### Moderated mediation effect analysis

**Figure 1.**
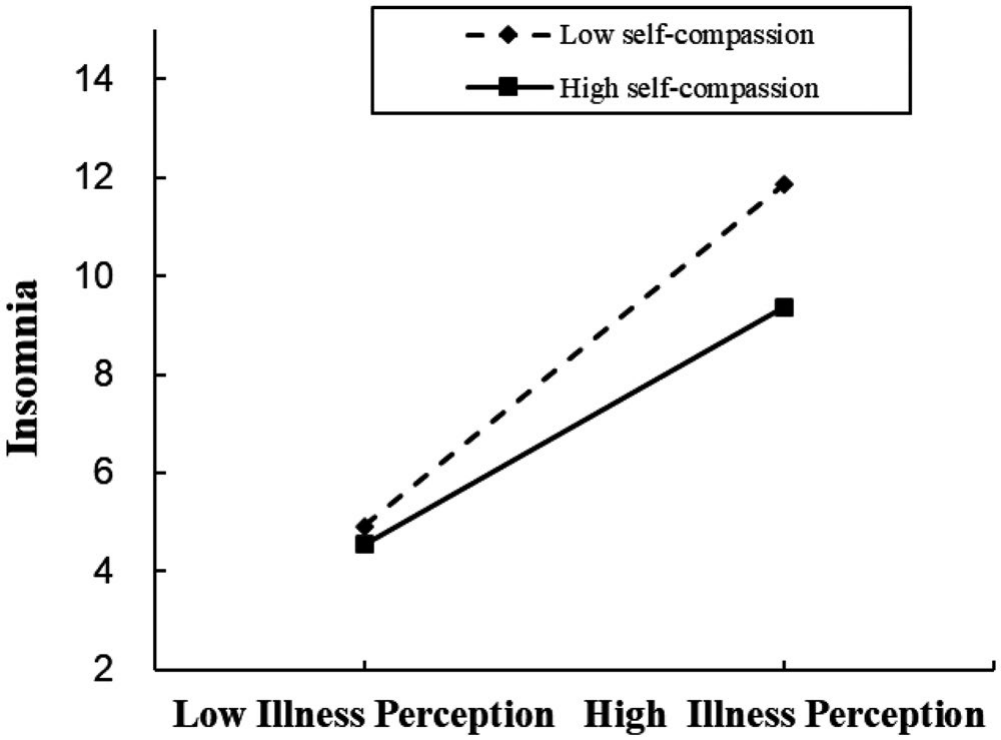
The moderating effect of self-compassion on the relation between fear of progression and insomnia.

Table 4 presents the results of the hierarchical multiple regression analysis examining the moderating effect of self-compassion on the illness perception-insomnia relationship. After controlling for all demographic and clinical covariates in Step 1, illness perception (BIPQ) was a significant positive predictor of insomnia in Step 2 (*B* = 0.364, *p* < 0.001, 95%CI [0.308, 0.414]), accounting for an additional 32.5% of the variance. Among the covariates, only BMI, medical payment methods, dialysis time and smoking were independently associated with insomnia severity in the fully adjusted model. The inclusion of self-compassion in Step 3 contributed a further 0.9% of explained variance (*B* = −0.114, *p* = 0.032, 95%CI [−0.207, −0.023]). Importantly, the interaction term BIPQ × SCS entered in Step 4 was statistically significant (B = −0.008, *p* = 0.030, 95%CI [−0.015, −0.001]), indicating that self-compassion significantly moderated the relationship between illness perception and insomnia. An analysis of simple slopes indicated that BIPQ was a predictor of insomnia at both low (*B* = 0.477, *t* = 5.131, and *p* < 0.001) and high (*B* = 0.732, *t* = 8.187, and *p* < 0.001) levels of self-compassion. When self-compassion levels were low, the relationship between BIPQ and insomnia became stronger (see Figure 1).

**Table 4.** Hierarchical multiple regression analyses of illness perception and self-compassion on insomnia (*n* = 332).

Steps and independent		Insomnia				
Variables	B (SE)	*β*	95% CI	*p*	Total *R*^2^	Δ*R*^2^
Step 1						
BMI	−0.646(0.313)	−0.655	[−1.261, −0.030]	0.040		
Medical payment methods	−1.831(0.487)	−1.804	[−2.789, −0.873]	<0.001		
Dialysis time	0.142(0.063)	0.682	[0.018, 0.266]	0.025		
Smoking	3.490(0.748)	3.001	[2.018,4.962]	<0.001	0.119	0.119
Step 2						
BIPQ	0.361(0.027)	0.364	[0.308,0.414]	<0.001	0.444	0.325
Step 3						
SCS	−0.115(0.047)	−0.114	[−0.207, −0.023]	0.014	0.453	0.009
Step 4						
BIPQ × SCS	−0.008(0.004)	−0.008	[−0.015, −0.001]	0.043	0.461	0.008

BMI: Body Mass Index; BIPQ, Brief Illness Perception Questionnaire; SCS, Self-compassion. All covariates were entered simultaneously in Step 1.

## Discussion

**Figure 2.**
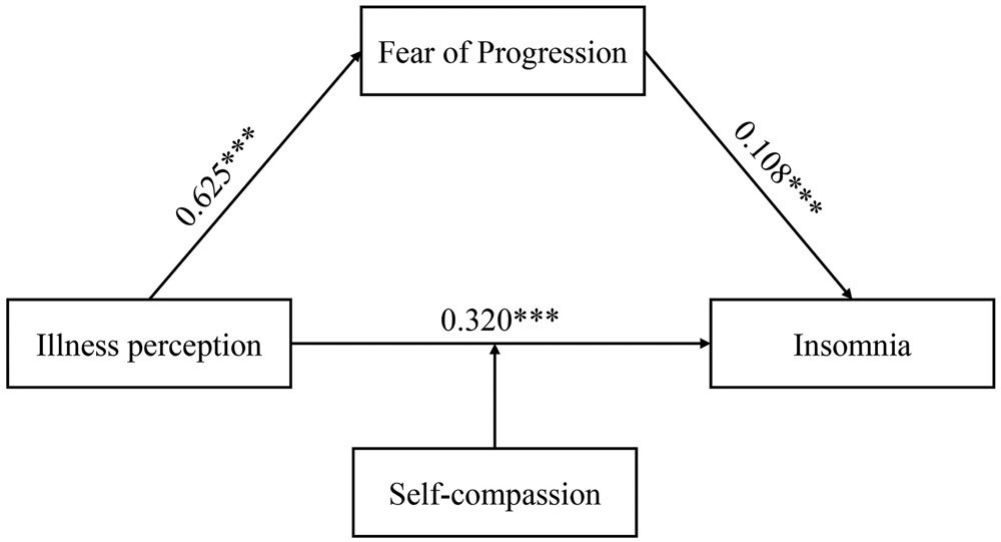
The final model with mediating effect and moderating effect (*n* = 332). Note: Mediation was tested using PROCESS Model 4; moderation of the direct effect was tested using hierarchical regression (PROCESS Model 1). ***p < 0.001.

This study explored the relationship between illness perception and insomnia in patients with maintenance hemodialysis, examining the mediating role of fear of progression and the moderating effect of self-compassion. Based on previous studies, we created a framework that negative illness perceptions indirectly influence insomnia through increased FoP, and this mediation process is buffered by higher levels of self-compassion (see Figure 2). These results contribute to deep understanding of the psychological mechanisms underlying sleep disturbances in MHD patients and align with broader literature on chronic disease management.

In this study, 55.72% of hemodialysis patients experienced insomnia. Consistent with previous research, our study confirms the high prevalence of insomnia among patients undergoing maintenance hemodialysis, which has been reported to affect 40–85% of this population [28,29]. The negative correlation between self-compassion and insomnia (*r* = −0.451) parallels the association between self-acceptance and insomnia (*r* = −0.531) reported by Tao et al. [28]. The study not only replicates these bivariate associations but also uniquely examines the underlying mechanisms and conditional effects. This is the initial research to explore fear of progression as a mediator in the connection between illness perception and insomnia in MHD patients, revealing that FoP contributes to 17.5% of this relationship. Furthermore, this study is the first to identify self-compassion as a significant moderator in the direct relationship between illness perception and insomnia (Δ*R*^2^ = 0.008, *p* = 0.030). Simple slope analyses further indicate that negative illness perceptions are more strongly associated with insomnia among patients with lower levels of self-compassion. Overall, these results enhance previous research by showing how illness perceptions can lead to sleep disturbance and identifying the individuals most affected, thus providing a more detailed and clinically relevant framework.

The link between medical payment methods and increased insomnia severity is consistent with existing research that emphasizes the mental impact of healthcare financial stress. Compared to employee insurance plans, resident health insurance often results in higher personal expenses and limited coverage for dialysis-related costs [30]. Our findings support that smoking is a significant predictor of insomnia in MHD patients, aligning with studies in the general population and those with chronic kidney disease [31]. A significant cross-sectional study utilizing NHANES data revealed that heavy smokers are 1.99 times more likely to report sleep issues than nonsmokers, showing a clear dose-response relationship [32].

The etiology of insomnia in MHD patients is attributed to a multifaceted interaction of biological factors, such as the presence of uremic toxins and disruptions in circadian rhythms, alongside psychological stressors [33,34]. Our findings further this comprehension by revealing that negative beliefs about disease control and consequences indirectly influence insomnia through fear of progression. This finding is consistent with meta-analytic evidence indicating that FoP serves as a transdiagnostic mediator, linking physical symptoms to adverse mental health outcomes in chronic illnesses [35].

The mediating influence of fear of progression needs to be highlighted. Fear of progression, characterized by an excessive fear of disease progression or recurrence, has been recognized as a significant source of distress in chronic conditions beyond oncology, such as renal disease [35]. Among hematological cancer survivors, FoP mediates the relationship between fatigue and quality of life [36], similar to our findings that FoP acts as a conduit between illness perception and insomnia in patients on maintenance hemodialysis. The observation indicates that FoP could serve as a universal psychological mechanism by which disease-related stressors result in sleep problems among various chronic illness populations.

The moderating effect of self-compassion introduces a critical protective mechanism into this model. Self-compassion is a significant personality characteristic and essential for mental well-being. Higher self-compassion correlates with improved mental health [37]. Self-compassion, involving self-kindness, mindfulness, and a feeling of shared humanity, might help decrease the tendency to negative illness perceptions, thereby reducing the increase of FoP. While there is limited direct evidence on self-compassion’s role in MHD, our findings align with existing studies indicating that self-compassion reduces the effects of psychological distress on health outcomes [38]. A 12-week meditation intervention study in hemodialysis patients showed that increased self-compassion was associated with reduced depressive symptoms, providing preliminary support for its mental health benefits in this population [39]. Our study provides insights into the potential of self-compassion to reduce sleep disturbances in people with MHD.

The incremental variance explained by the interaction term between the BIPQ and SCS (Δ*R*^2^ = 0.008) aligns with established literature on continuous moderator effects in field research. Median Δ*R*^2^ values for interaction terms in such studies typically range from 0.002 to 0.010 [40,41]. Furthermore, the substantial main effect of illness perception (*R*^2^ = 0.325) limits the residual variance available for the interaction to explain. Despite its modest absolute size, the interaction effect is statistically significant (95% CI [−0.015, −0.001]) and possesses meaningful clinical implications. Simple slope analysis indicated that among patients with low self-compassion, illness perception was more strongly associated with insomnia severity. This finding suggests that self-compassion serves as a protective psychological resource and reduces the adverse sleep consequences of negative illness perception. Given that self-compassion can be effectively improved through mindful self-compassion exercises, even a modest moderating effect may translate into meaningful clinical benefits when applied in practice.

The underlying mechanisms can be interpreted from both psychological and physiological perspectives. Psychologically, Negative illness perception elevates threat‑related cognitive appraisal, which further increases fear of disease progression. This chain of reactions leads to persistent hyperarousal, rumination, and anxiety, all of which are core mechanisms in the onset and maintenance of insomnia. Physiologically, persistent psychological stress can activate the hypothalamic-pituitary-adrenal (HPA) axis and raise cortisol levels. These physiological changes disrupt circadian rhythms and decrease melatonin secretion, directly impairing sleep structure and continuity. Self-compassion may buffer this pathway by reducing over-identification with negative thoughts, lowering stress reactivity, and restoring emotional regulation.

These results have obvious and practical implications for clinical practice. First, routine screening for illness perception, fear of progression, and self-compassion levels should be integrated into standard care for MHD patients. Second, targeted psychological interventions can be carried out. Cognitive restructuring to correct negative illness beliefs and reduce catastrophic thinking about disease progression. Brief mindfulness and self-compassion training to enhance emotional coping and reduce sleep-related hyperarousal. Integrated cognitive-behavioral therapy for insomnia (CBT-I) adapted for dialysis patients, combining sleep hygiene education with psychological support [42,43]. Third, teams composed of nephrologists, nurses, and clinical psychologists should work together to offer tailored psychological and sleep management, enhancing sleep quality and long-term results.

## Limitations

There are several limitations in this study that need to be considered. Firstly, due to the study’s cross-sectional design, we cannot definitively determine the direction of the associations observed. While our theoretical model suggests that illness perception comes first and fear of progression acts as a mediator, it’s possible that insomnia could worsen negative illness perceptions or heighten fear of progression through mechanisms such as hyperarousal and cognitive bias. A significant indirect effect found in the mediation analysis implies that the data support the hypothesized mediational pathway, but it does not establish causality. Different directional models could be equally valid. Additionally, cross-sectional estimates of indirect effects can be inaccurate relative to longitudinal estimates, especially when the underlying processes occur over long durations. Thus, the mediation effect mentioned should be seen as an associative breakdown rather than a conclusive causal measure. Repeated measurements in longitudinal studies are crucial for establishing temporal precedence and confirming the suggested causal pathways. Secondly, even though we accounted for various demographic and clinical factors, we did not gather data on sleep-disordered breathing, restless legs syndrome or detailed medication profiles. These conditions are very common among MHD patients and might independently lead to insomnia. The primary adjusted models did not include dialysis adequacy (Kt/V) and biochemical parameters like hemoglobin and serum calcium. To reduce unmeasured confounding, future research should include objective sleep evaluations and detailed clinical phenotyping. Thirdly, the design of the study being limited to a single center restricts its geographic and institutional applicability. Studies conducted across multiple centers with varied samples are needed. Fourthly, using self-report measures might lead to common method bias, but employing validated tools with strong internal consistency helps to alleviate this issue. Fifthly, the primary mediation analysis (PROCESS Model 4) was conducted without the inclusion of demographic or clinical covariates. The choice of analysis was made to align structurally with the moderated mediation model (PROCESS Model 5). Since initial bivariate analyses showed that only smoking status and the method of medical payment were significantly linked to insomnia in our study. Sixthly, even though our main analyses were based on hypotheses, we did not use formal adjustments for multiple comparisons within the correlation matrix. Hence, the bivariate associations in Table 2 require careful interpretation. Further studies with larger, independent samples are needed to confirm the observed relationships. Finally, this study excluded patients with diagnosed major psychiatric disorders. This exclusion criterion was applied to reduce the impact of severe mental disorders and to maintain the accuracy of self-reported data. Nonetheless, we recognize that this decision may lead to selection bias. Consequently, the observed prevalence of insomnia (55.72%) and the effect sizes for the psychological associations reported herein may be conservative estimates of the true population parameters. The connection between illness perception and insomnia might be stronger in patients with both depression or anxiety, who were not included in this study. Future research should involve patients with stable, treated psychiatric disorders and employ validated measures for depression and anxiety to more accurately define the psychological risks linked to MHD-related insomnia.

## Conclusions

This research enhances the comprehension of insomnia in MHD patients by discovering a moderated mediation pathway that includes illness perception, fear of progression, and self-compassion. There is a positive association between illness perception and insomnia, with fear of progression mediating this relationship, and self-compassion playing a moderating role. These results highlight the need to consider psychological aspects in addition to medical treatment in MHD care. Our research supports the adoption of psychological methods that aim to altering illness perceptions, lessen fear of progression, and enhance self-compassion. Future longitudinal studies are needed to determine causality and assess the effectiveness of CBT-I interventions combined with self-compassion training. Healthcare providers can enhance the sleep quality and overall well-being of MHD patients by focusing on these psychological mechanisms.

## Data Availability

Upon a reasonable request, the datasets employed and examined in this study can be accessed through the corresponding author.
